# Supplementary material for: Preservation of protein expression systems at elevated temperatures for portable therapeutic production
Source: J R Soc Interface. 2017 Apr 26;14(129):20161039. doi: 10.1098/rsif.2016.1039 (PMC5414909; doi:10.1098/rsif.2016.1039)
Supplement: Supplementary Information [file rsif20161039supp1.pdf]

Supplementary Materials

Preservation of protein expression systems at elevated temperatures for portable therapeutic production

David K. Karig[*1,2], Seneca Bessling[1], Peter Thielen[1], Sherry Zhang[1], Joshua Wolfe[1]

[1]Research and Exploratory Development Department, Johns Hopkins University Applied Physics Laboratory, Laurel, MD, USA

[2]Department of Chemical and Biomolecular Engineering, Johns Hopkins University, Baltimore, MD, USA

[*]Correspondence and requests for materials should be addressed to David Karig
E-mail: David.Karig@jhuapl.edu; Tel.: 240-228-4719, Fax: 443-778-3500

Supplementary Results

Supplementary Figure 1 – Comparison of commercial and homemade cell-free systems

Supplementary Figure 2 – Effect of trehalose on expression

Supplementary Figure 3 – Dried reaction buffer variants

Supplementary Figure 4 – Exploring omission of different reaction buffer components

Supplementary Figure 5 – Creatine phosphate stability

Supplementary Figure 6 – Effect of trehalose on protein product

Supplementary Figure 7 – Long-term storage experiment with all components preserved

Supplementary Figure 8 – Percentage yield retention vs. storage time for Figure 2 experiments

Supplementary Figure 9 – Minimum inhibitory concentrations vs. storage time

Supplementary Table 1 – Summary of yield estimates

Supplementary Note 1 – Reaction buffer variants

Supplementary Note 2 – Pyocin killing analysis

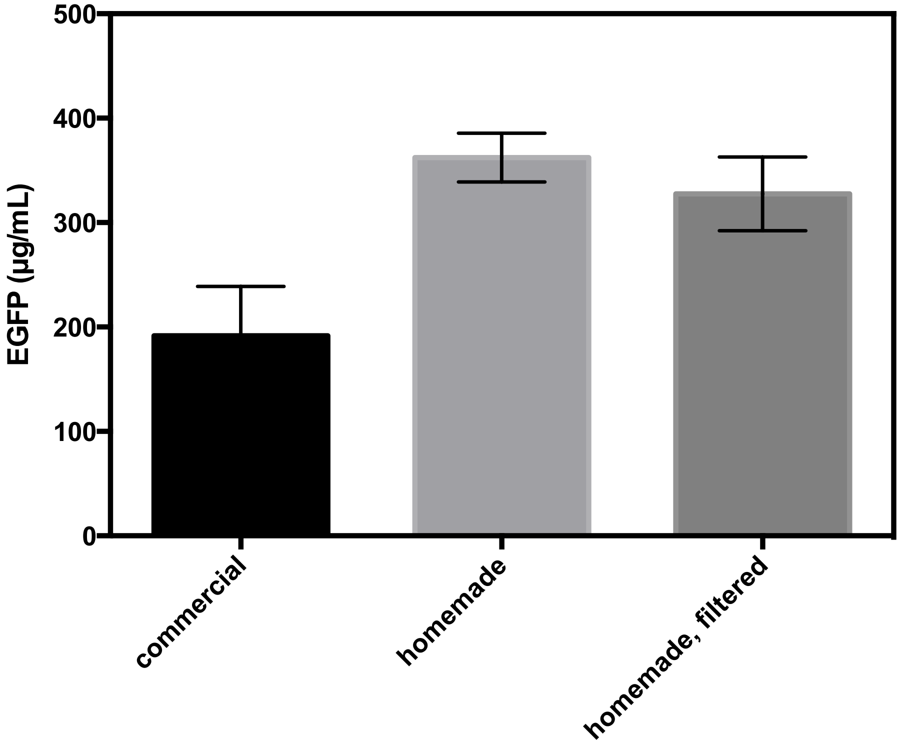

Figure SI1: Yield quantification. To provide a frame of reference for normalized fluorescence values, we compared the commercially available Promega S30 T7 High-Yield Expression System to extract and reaction buffer produced in house as described in the Methods section. We also quantified the effect of filtration on expression. To test expression, the pUCT7tet-T7term expression construct was added to each set of cell-free expression reagents, and fluorescence resulting from EGFP expression was measured using a plate reader. Relative Fluorescence Units (RFU) from the plate reader were recorded after 5 h of incubation. Background correction was performed by subtracting fluorescence readings from reactions with no DNA. Columns represent the mean of four measurements, and error bars depict standard deviation. Expression from homemade cell-free reagents was found to be 75% greater than with the commercial kit. Expression levels from non-filtered and filtered reagents were within one standard deviation of one another. To calculate yield from plate reader fluorescence measurements, we first purified EGFP as previously described[1] and quantified protein concentration using a Pierce BCA Protein Assay kit. We then added different dilutions of purified EGFP to cell-free reaction mixture and measured fluorescence intensities on the plate reader to create a calibration curve.

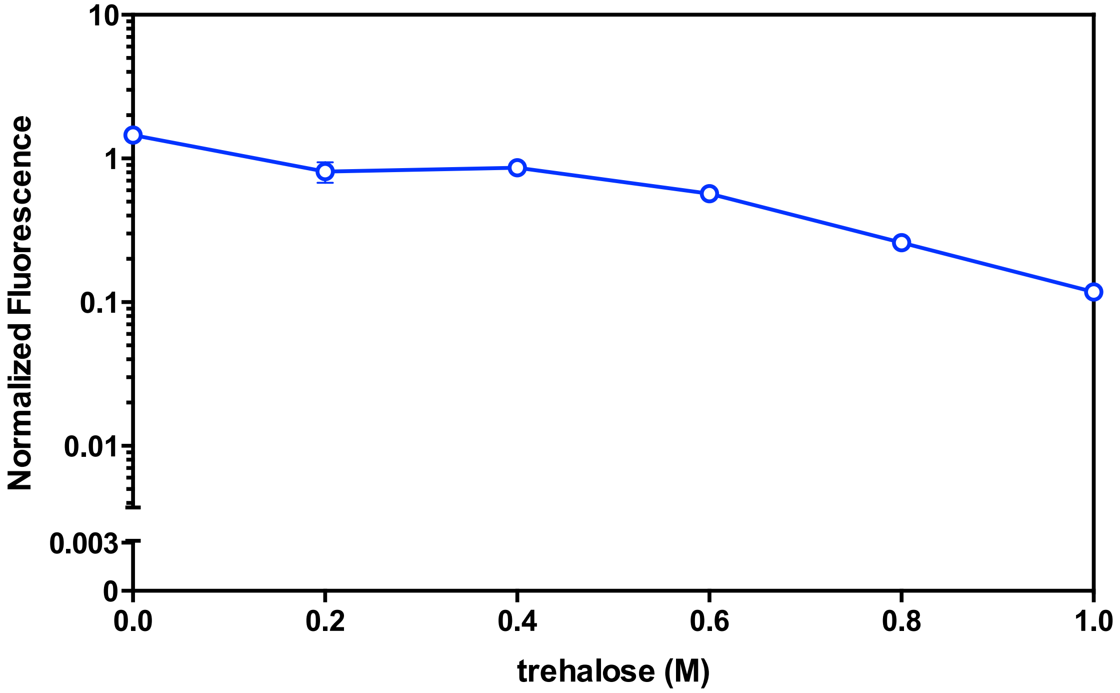

Figure SI2: Effect of trehalose on expression. EGFP expression reactions were set up using different concentrations of trehalose. Normalized fluorescence after 5 h of incubation is shown. All data points represent the mean of triplicate measurements, and error bars depict standard deviation. Cases where no error bars are visible indicate that error bars are smaller than the marker.

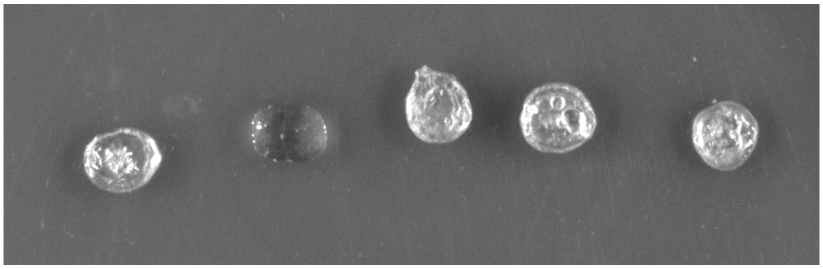

Figure SI3: Dried reaction buffer variants. Each reaction buffer was prepared with 0.6 M trehalose and omitting magnesium acetate and creatine phosphate. From left to right, additional components were omitted prior to drying: amino acids, PEG, DTT, creatine kinase, and no additional omissions. Aliquots of 35 μL were dried. The distinctly clear, glasslike appearance of dried buffer lacking PEG prompted further exploration of the effect of PEG on stability (Figure 2d-e).

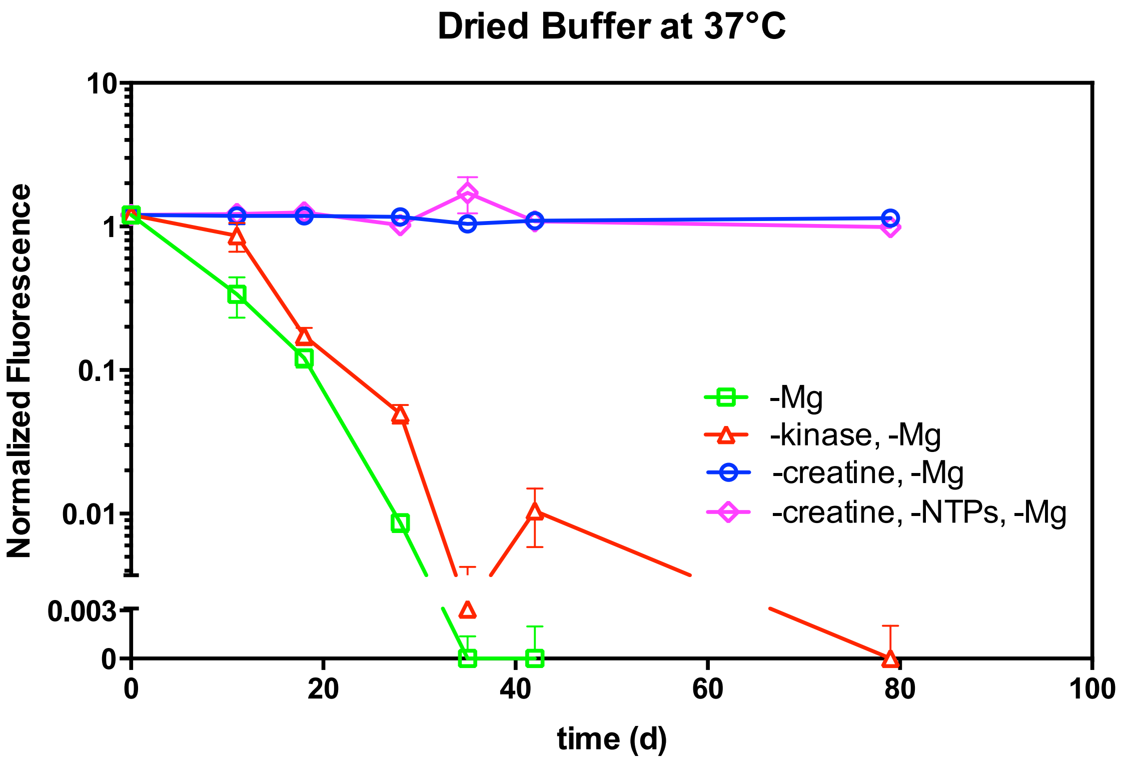

Figure SI4: Examples of experiments exploring the omission of different reaction buffer components. Although we explored the omission of several components, we found that creatine phosphate omission had the most significant effect. We show four examples here to highlight the benefit of separately storing creatine phosphate. Four variants of reaction buffer were prepared with 0.54 M trehalose and dried: 1) omitting magnesium acetate (green squares), 2) omitting magnesium acetate and creatine kinase (red triangles), 3) omitting magnesium acetate and creatine phosphate (blue circles), and 4) omitting magnesium acetate, nucleotide triphosphates, and creatine phosphate (purple diamonds). At different time points during storage at 37°C, dried reaction buffer variants were reconstituted with water, and components originally omitted from each reaction buffer variant were re-added. Reconstituted buffer and fresh extract were mixed. Expression capacity was then assessed by adding a T7-EGFP expression construct and measuring fluorescence after 5 h of incubation. For the two reaction buffer variants that included creatine phosphate, 100-fold decreases in expression capacity were observed. By contrast, for the two variants in which creatine phosphate was omitted, no significant reduction in expression capacity was observed for the duration of the experiment. Thus, separately storing creatine phosphate from other reaction buffer ingredients improves stability. All data points represent the mean of triplicate measurements, and error bars depict standard deviation. Cases where no error bars are visible indicate that error bars are smaller than the marker.

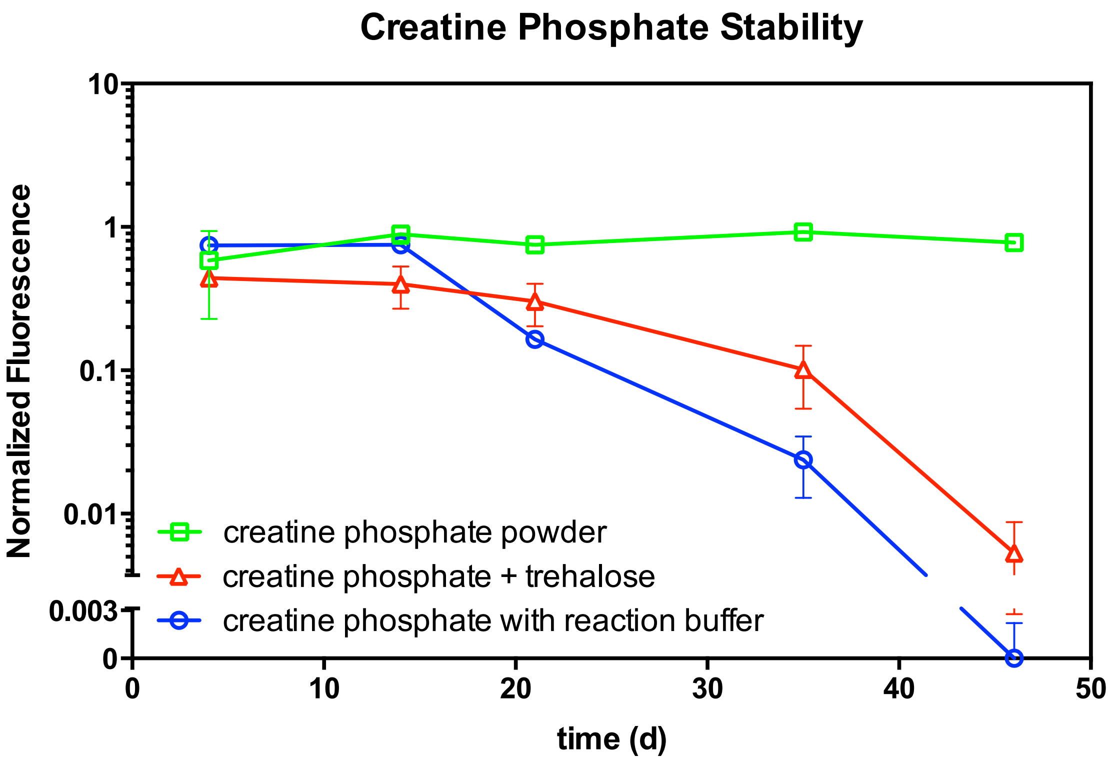

Figure SI5: If the energy source, e.g. creatine phosphate, is to be stored separately, its stability at 37°C must be ensured. An experiment was conducted to examine the stability of creatine phosphate in dry form and creatine phosphate mixed with trehalose in liquid form and dried. Creatine phosphate powder, creatine phosphate dried with 0.6 M trehalose, and creatine phosphate with reaction buffer (omitting magnesium acetate) dried with 0.53 M trehalose were stored at 37°C. At different time points during storage at 37°C, full reaction buffers were prepared from the heat stressed components. Specifically, creatine phosphate powder was combined with fresh reaction buffer reagents and 0.53 M trehalose. Creatine phosphate dried with trehalose was combined with fresh reaction buffer reagents and 0.48 M trehalose. Dried reaction buffer was reconstituted with water and combined with magnesium acetate. Note that trehalose concentrations in the fresh reaction buffer were chosen to keep the final reaction concentration the same for each sample. Each of these reaction buffers was then combined with fresh extract. Expression capacity was then assessed by adding a T7-EGFP expression construct and measuring fluorescence after 5 h of incubation. When creatine phosphate was stored separately in powder form and without trehalose, no reduction in expression capacity was observed for the duration of the experiment. Therefore, additives such as trehalose are unnecessary and can in fact be detrimental to stability. All data points represent the mean of triplicate measurements, and error bars depict standard deviation. Cases where no error bars are visible indicate that error bars are smaller than the marker.

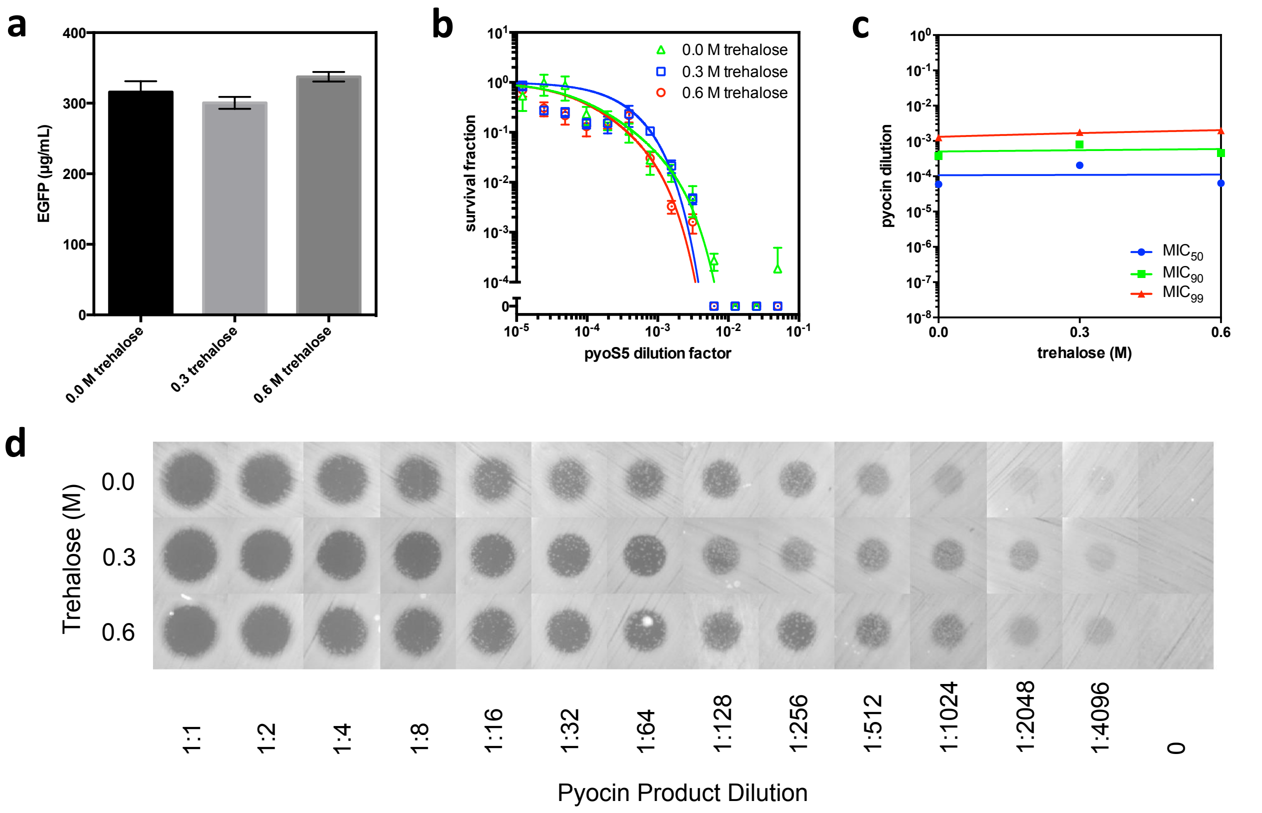

Figure SI6: Effect of trehalose on protein product. EGFP and pyocin S5 were separately expressed using fresh cell-free reagents. After 5 hours of expression at 30°C, trehalose was added at the indicated concentrations to the final EGFP and pyocin S5 reactions. **a)** Effect on fluorescence resulting from GFP expression. **b)** Broth dilution assay to quantify pyocin killing efficacy. **c)** MIC values derived from broth dilution data with linear regression fits. **d)** Pyocin clearing assay.

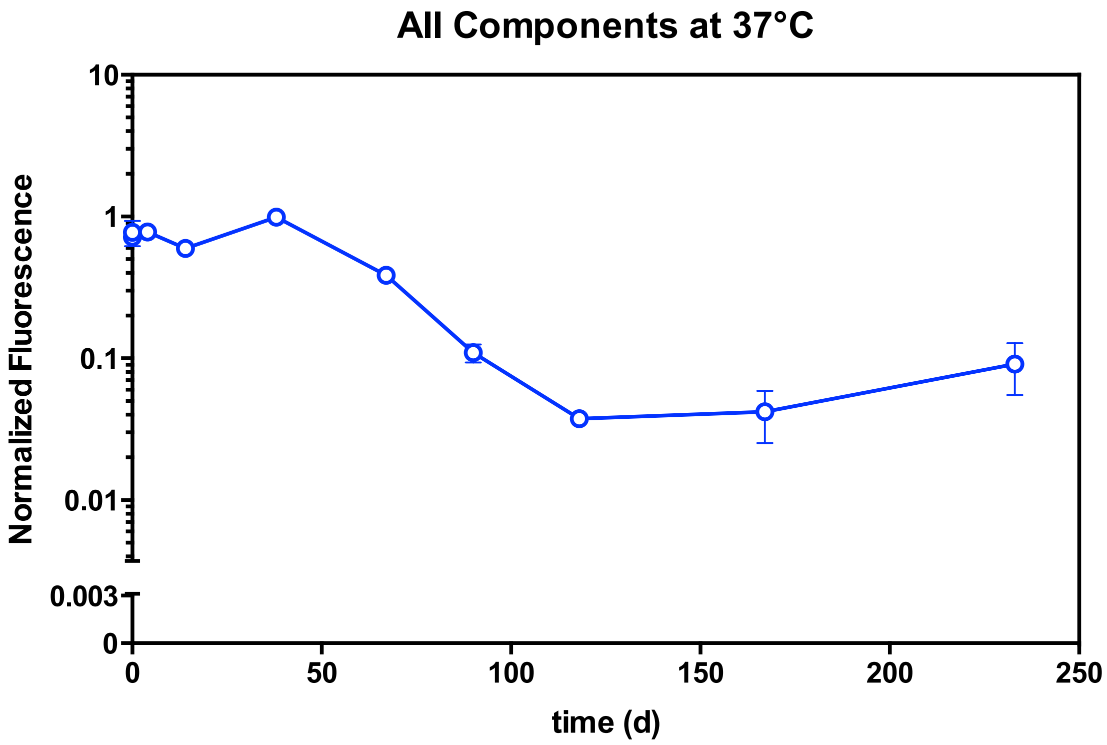

Figure SI7: Long-term storage experiment with all components preserved. Cell extract dried with 0.54 M trehalose, reaction buffer (omitting creatine phosphate and magnesium acetate) dried with 0.59 M trehalose, creatine phosphate powder, and magnesium acetate were all stored at 37°C under atmospheric conditions. At different time points during storage at 37°C, components were reconstituted and combined to create an expression reaction mixture. Expression capacity was then assessed by adding a T7-EGFP expression construct and measuring fluorescence after 5 h of incubation. After approximately two months, expression capacity decreased to a lower plateau. However, even after eight months at 37°C, significant expression over background was observed, as fluorescence levels were always at least ten times greater than the background threshold. All data points represent the mean of triplicate measurements, and error bars depict standard deviation. Cases where no error bars are visible indicate that error bars are smaller than the marker.

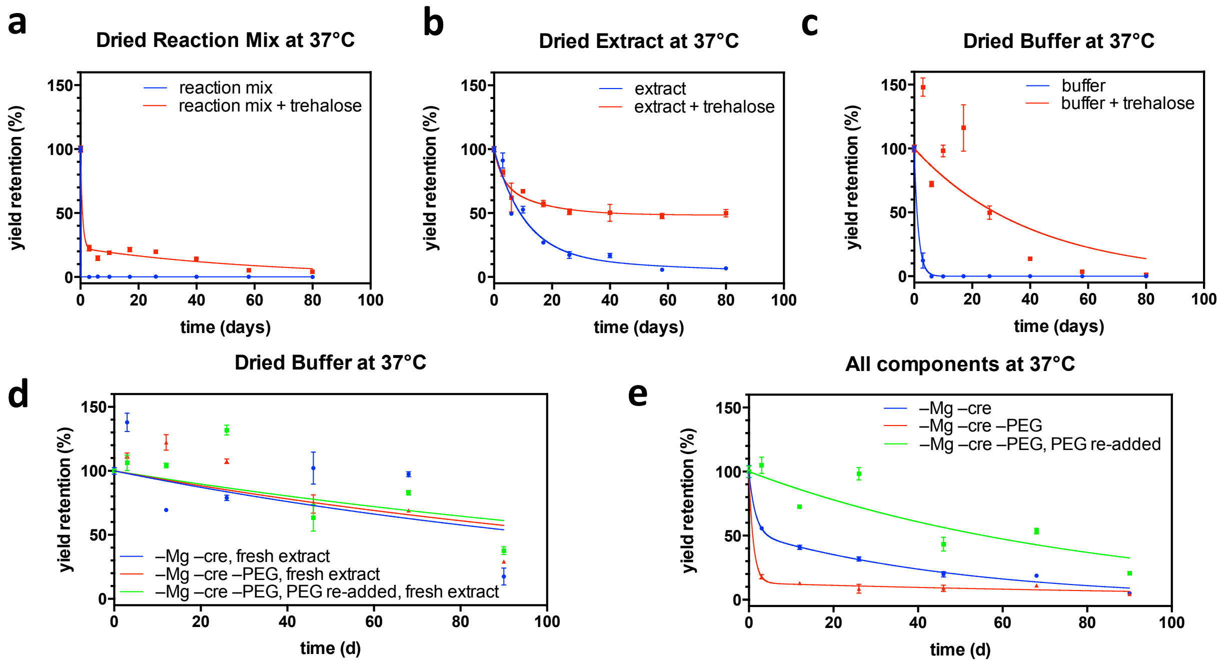

Figure SI8: Percentage yield retention vs. storage time for Figure 2 experiments. Each curve is normalized to its day 0 value such that all curves begin at 100%. All data points represent the mean of triplicate measurements, and error bars depict standard deviation. Cases where no error bars are visible indicate that error bars are smaller than the marker. **a)** Reaction mixture dried with and without trehalose and stored at 37°C. **b)** Cell extract dried with and without trehalose, stored at 37°C, and tested using fresh reaction buffer. **c)** Reaction buffer dried with and without trehalose, stored at 37°C, and tested with fresh cell extract. **d)** Reaction buffer variants dried with trehalose, stored at 37°C, and tested with fresh cell extract. In all cases, magnesium acetate and creatine phosphate were stored separately at 37°C and were combined with reactions at the time of reconstitution and testing. Variations involved including PEG in the reaction buffer before drying, omitting PEG from the reaction buffer, and storing PEG separately for addition at the time of reconstitution and testing. **e)** Same conditions as (d), but using extract dried with trehalose and stored at 37°C. In other words, all expression reaction components were stored at 37°C.

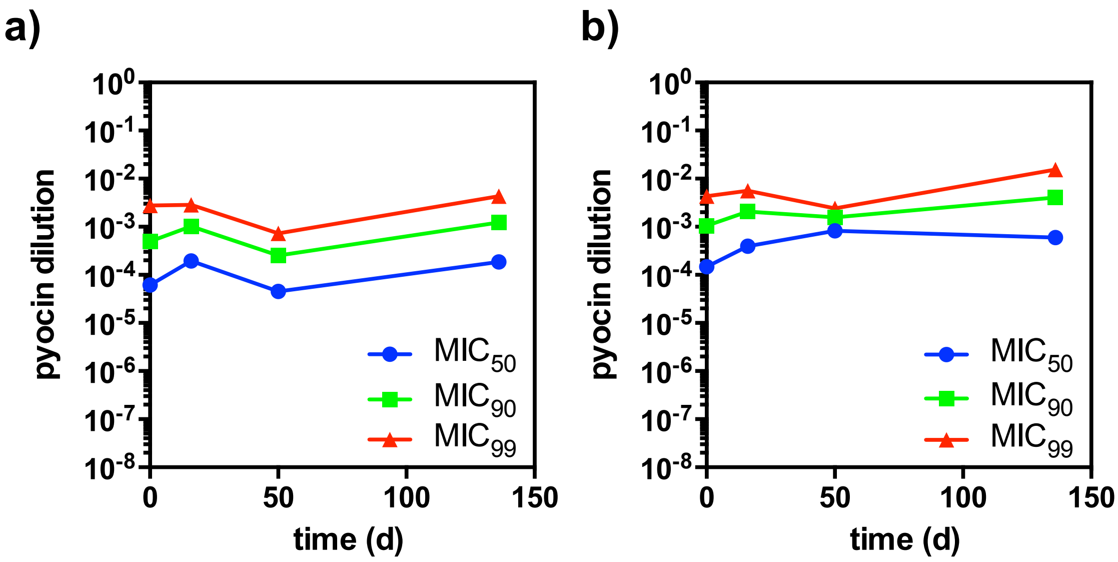

Figure SI9: Minimum inhibitory concentrations (MIC_{50}, MIC_{90}, and MIC_{99}) were calculated using concentration-killing curve fits for the broth dilution experiments in **a)** Figure 3b and **b)** Figure 3d. For both experiments, all MIC values are within an order of magnitude for the full duration of the 136 d experiment.

Table 1

extract	reaction buffer	additional reagents (stored separately at 37°C)	normalized yield	absolute yield (µg/mL)	yield retention (%)
Combined with reaction buffer, stored at 37°C	Combined with extract, stored at 37°C	--	0*	0*	0*
Stored at 37°C	Stored at -80°C	--	0*	0*	0*
Stored at -80°C	Stored at 37°C	--	0*	0*	0*
Combined with reaction buffer, dried, stored at 37°C	Combined with extract, dried, stored at 37°C	Magnesium acetate	0*	0*	0*
Combined with reaction buffer and trehalose, dried, stored at 37°C	Combined with extract and trehalose, dried, stored at 37°C	Magnesium acetate	0.06	19	9
Dried, stored at 37°C	Stored at -80°C	Magnesium acetate	0.08	25	8
Dried with trehalose, stored at 37°C	Stored at -80°C	Magnesium acetate	0.29	89	49
Stored at -80°C	Dried, stored at 37°C	Magnesium acetate	0*	0*	0*
Stored at -80°C	Dried with trehalose, stored at 37°C	Magnesium acetate	0.15	46	23
Stored at -80°C	Dried with trehalose, stored at 37°C	Magnesium acetate, creatine phosphate	0.47	144	66
Stored at -80°C	PEG omitted, dried with trehalose, stored at 37°C	Magnesium acetate, creatine phosphate	0.25	76	69
Stored at -80°C	PEG omitted, dried with trehalose, stored at 37°C	Magnesium acetate, creatine phosphate, PEG	0.52	156	72
Dried with trehalose, stored at 37°C	Dried with trehalose, stored at 37°C	Magnesium acetate, creatine phosphate	0.07	21	16
Dried with trehalose, stored at 37°C	PEG omitted, dried with trehalose, stored at 37°C	Magnesium acetate, creatine phosphate	0.06	18	8
Dried with trehalose, stored at 37°C	PEG omitted, dried with trehalose, stored at 37°C	Magnesium acetate, creatine phosphate, PEG	0.09	29	47

*No detectable product, as fluorescence is indistinguishable from background.

Table 1: Yield estimates after 60 days of storage for experiments in Figures 1-2. Estimates at 60 days were derived from two-phase decay fits, as shown and described in Supplementary Figure 8. Absolute yield was based on calibration of plate reader fluorescence measurements to a standard curve generated using purified EGFP (see Supplementary Figure 1). Yield retention was calculated from the yield at 60 days, divided by the initial yield measurement.

Supplementary Note 1

We explored several reaction buffer variants, including omission of creatine phosphate (Fig. 2d), creatine kinase, and NTP's (Supplementary Fig. 4). In addition, motivated by the fact that omitting PEG resulted in dried reaction buffer aliquots that were clear and glassy in appearance by comparison to other formulations (Supplementary Fig. 3), we examined the effects of omitting PEG, both with and without re-addition after storage and reconstitution. Over the course of the 37°C exposure experiment, expression remained lower for the sample in which PEG was omitted and not added upon reconstitution (Fig. 2d). Interestingly, however, whether or not PEG was included in the reaction buffer before drying had little effect, despite its impact on the appearance of the dried buffer.

Supplementary Note 2

In order to quantitatively analyze killing efficiency over time, we fit broth dilution data to a concentration-killing curve [2]. Fits are shown in Figure 3b,d:

$$N = \frac{N_0}{1 + e^{r(x-B)}}$$

Here, *N* is the fraction of surviving cells, *x* is the pyocin concentration, *r* captures the steepness of the dosage response, and *B* is the pyocin dosage yielding half-maximal killing. Using the fits, we quantified the effective minimum inhibitory concentrations (MIC50, MIC90, and MIC99) in Matlab. As shown in Supplementary Figure 9, these MIC values ranged within an order of magnitude for the full duration of the 136 d experiment. To offer a point of reference, the lowest characterized MIC of pyocin S5 for a *P. aeruginosa strain* to date is 12.6 µg/mL[3].

References

1. Retterer, S.T., Siuti, P., Choi, C.-K., Thomas, D.K. & Doktycz, M.J. Development and fabrication of nanoporous silicon-based bioreactors within a microfluidic chip. *Lab on a Chip* 10, 1174-1181 (2010).
2. Liu, Y., Zhang, Y. & Gao, P. Novel concentration-killing curve method for estimation of bactericidal potency of antibiotics in an in vitro dynamic model. *Antimicrobial agents and chemotherapy* 48, 3884-3891 (2004).
3. Ling, H., Saeidi, N., Rasouliha, B.H. & Chang, M.W. A predicted S-type pyocin shows a bactericidal activity against clinical Pseudomonas aeruginosa isolates through membrane damage. *FEBS Lett* 584, 3354-3358 (2010).
